# Frequency domain photoacoustic and fluorescence microscopy

**DOI:** 10.1364/BOE.7.002692

**Published:** 2016-06-20

**Authors:** Gregor Langer, Bianca Buchegger, Jaroslaw Jacak, Thomas A. Klar, Thomas Berer

**Affiliations:** 1 Research Center for Non-Destructive Testing GmbH, Altenberger Straße 69, 4040 Linz, Austria; 2 Institute for Applied Physics, Johannes Kepler University Linz, Altenberger Straße 69, 4040 Linz, Austria; 3 University of Applied Sciences Upper Austria, Applied Health & Social Sciences, Garnisonstraße 21, 4020 Linz, Austria

**Keywords:** (170.5120) Photoacoustic imaging, (180.2520) Fluorescence microscopy, (110.5125) Photoacoustics, (170.3880) Medical and biological imaging, (140.2020) Diode lasers

## Abstract

We report on simultaneous frequency domain optical-resolution photoacoustic and fluorescence microscopy with sub-µm lateral resolution. With the help of a blood smear, we show that photoacoustic and fluorescence images provide complementary information. Furthermore, we compare theoretically predicted signal-to-noise ratios of sinusoidal modulation in frequency domain with pulsed excitation in time domain.

## 1. Introduction

Fluorescence microscopy became a valuable tool for life sciences within the last century. Although many groups of molecules exhibit fluorescence, most do not. To allow fluorescence imaging of non-fluorescent molecules, these have to be tagged by fluorophores [1]. However, fluorescent tagging of samples is not always an option as it might change biological or chemical processes [2]. In general, all molecules are optically absorbing at some wavelengths, even when they do not exhibit luminescence. When the optically-excited molecules return into their ground state, the absorbed energy is either emitted radiatively via luminescence and/or is transferred into heat via non-radiative processes. The latter process leads to thermal expansion and to the generation of ultrasonic waves, commonly known as the photoacoustic effect [3]. By simultaneously employing fluorescence and photoacoustic imaging, both relaxation processes are monitored. Thereby valuable complementary information can be obtained which could be missed if only one of both methods is applied. Simultaneous photoacoustic and fluorescence imaging was, e.g., demonstrated by Wang et al. [4].

In the present paper, we introduce a frequency domain optical-resolution photoacoustic microscope (fOR-PAM) in combination with a frequency domain fluorescence microscope. Commonly in optical-resolution photoacoustic microscopy (OR-PAM), short laser pulses are focused into a specimen to generate photoacoustic signals at the optical focus. Microscale resolution is obtained by scanning the focused laser spot [5–12]. In OR-PAM, the lateral resolution is intrinsically determined by the excitation laser’s beam waist, and a photoacoustic resolution as small as 0.22 µm was demonstrated [6]. By making use of non-linear effects, even better resolutions have been realized [13,14]. For pulsed excitation, the axial resolution, which depends on a time-of-flight measurement, is determined by the bandwidth of the ultrasonic transducer and is, therefore, typically larger than 10 µm [4,8,15]. This results in highly asymmetric voxels, with the lateral resolution typically being more than 10-times better than the axial. As an alternative to time domain photoacoustic imaging, frequency domain photoacoustic imaging was demonstrated [16–20]. Sources for frequency domain imaging may be more cost effective, come with smaller footprints [19], and require less maintenance compared to the typically employed Q-switched ns-lasers. In comparison to time domain excitation, the generation of acoustic pressure waves is usually much less effective for frequency domain excitation. To compensate for this, pulse compression techniques using frequency chirps and matched filtering [16,17,21] are frequently employed. In the present paper, we excite photoacoustic and fluorescence signals using a cw diode laser with sinusoidal amplitude modulation. In photoacoustic imaging (PAI), this can be considered as the limiting case of a “chirped” excitation with a single frequency and vanishing detection bandwidth. Thereby, all axial information is lost. This is not necessarily a disadvantage as, e.g., microtome cuts and eukaryotic cells typically have thicknesses up to 10 µm. For these kinds of specimens, time domain investigation does not provide sufficient axial resolution to gain additional information anyhow. In some way, the proposed method is related to the techniques used at the very advent of photoacoustic microscopy were cw lasers were chopped and the resulting photothermal wave signals were measured with the help of microphones and lock-in techniques [22,23]. A very similar technique was introduced to fluorescence microscopy more than a decade ago (see e.g [24].). By measuring amplitude and phase of the fluorescence signal in frequency domain, it is not only possible to create fluorescence intensity images of samples, but also to determine the lifetimes of fluorophores [24–26].

## 2. Setup

**Fig. 1:**
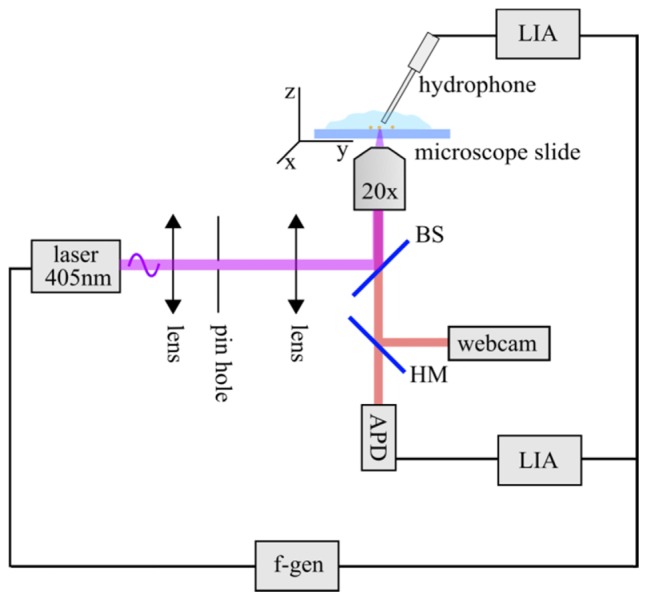
Schematic of the setup. A function generator (f-gen) is used to modulate a cw diode laser (laser 405 nm). Laser light is focused onto the sample by an objective lens (20 × , NA = 0.5). Photoacoustic and luminescence signals are measured via a hydrophone and an avalanche photo-diode (APD), respectively. The lock-in amplifiers (LIA) are set to the modulation frequency. The 3D scanning stage is symbolized by the xyz coordinate system.

A schematic of the setup is shown in Fig. 1

. Generation of the photoacoustic and luminescence signals is performed with a diode laser with a wavelength of 405 nm (Omicron, LuxX + 405-120). The output power of the diode laser is sinusoidally modulated by a function generator (Agilent 33250A). The diode laser has a maximum cw power of 120 mW and exhibits a 3 dB decrease in modulation depth at around 3 MHz. This restricts the modulation frequencies to the low MHz regime. The excitation laser beam is expanded and mode cleaned via a telescope and a 30 µm aperture (Edmund Optics, copper high power pin hole). It is then focused onto the specimen via a 20 × objective lens (Olympus, UPlanFl 20 × , NA = 0.5, corrected for 0.17 mm cover slips). Samples to be analyzed are fixed on silanized microscope slides with a thickness of 1 mm (Superior Marienfeld, Histobond) and immersed in deionized water in order to ensure acoustic coupling to the hydrophone (Onda, needle hydrophone: HNC 1000, preamplifier: AH-2020). The microscope slides are fixed on a three-axis nanopositioning stage (Physik Instrumente, PIMars P563.3CD). Luminescence light is collected by the objective lens and passes through a beam splitter BS (Semrock, quadband beam splitter) and a hot mirror HM (Thorlabs, red hot mirror: FM02) before being detected by an avalanche photo diode (APD, Hamamatsu, C12702-11). The hydrophone and the APD are connected to two lock-in amplifiers (LIA, Stanford Research, SR844), locked at the modulation frequency provided by the function generator. The measurement data is stored within the lock-in amplifier internal memories. Due to experimental limitations, i.e. lock-in data storage time, laser modulation, and frequency dependence of the hydrophone’s sensitivity, we used lock-in integration times between 10 ms and 30 ms and modulation frequencies between 4 MHz and 10 MHz. After the scan, the data is transferred to a computer via a GPIB-USB interface (Prologix). A webcam (Logitech, C300) is used to get bright-field microscopic images of the sample.

## 3. Measurements

### 3.1 Determination of the excitation spot diameter

**Fig. 2:**
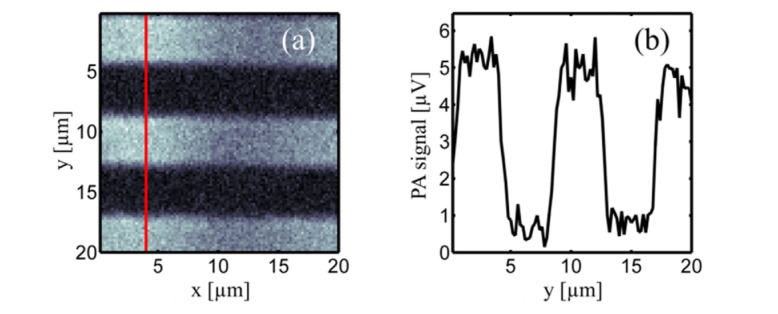
(a) fOR-PAM image of a chromium line target with 120 lines/mm. (b) Profile of the photoacoustic image along the red line.

The excitation spot diameter was evaluated with the aid of a chromium line target (Edmund Optics, resolution line target, 120 lines/mm). Figure 2(a)

shows a photoacoustic scan over an area of 20 µm × 20 µm consisting of 101 × 101 pixels. The modulation frequency of the excitation laser was 4 MHz and the excitation power was ~8 mW. The time required for the acquisition of the image was approximately 100 s. In Fig. 2(b), the profile along the red line in Fig. 2(a) is shown. By assuming that the photoacoustic resolution is solely determined by the laser beam profile and that the laser profile is of Gaussian shape, one can fit the slopes in Fig. 2(b) by an error function, erf(*y*). From the argument of the error function, we calculated the full-width-at-half-maximum (FWHM) of the excitation laser beam waist to be 0.75 ± 0.1 µm.

In case of truncated Gaussian beams, the diffraction limited 1/e^2^ beam diameter *s* in the focal plane can be estimated by [27]

$$s\approx \frac{K\;\text{λ}}{2\;\text{NA}},$$ (1)

where λ is the light’s wavelength and NA is the numerical aperture of the objective. *K* is a factor that describes the Gaussian beam truncation at the objective for a given 1/e^2^ beam diameter *w* and the diameter of the objective’s entrance pupil *a* [27]

$$K=1.654-\frac{0.105\;a}{w}+\frac{0.28\;a^{2}}{w^{2}}.$$ (2)

In the presented setup, the diode laser emits a Gaussian beam with a 1/e^2^ beam diameter of 1.25 ± 0.25 mm [28]. The laser beam is expanded via a telescope by a factor of 15/4, leading to a 1/e^2^ beam diameter of 4.69 ± 0.94 mm before the objective’s entrance pupil. With an entrance pupil diameter of 9 mm, *K* is approx. 2.5. From Eq. (1) and considering that the FWHM is a factor of $\sqrt{2/\ln(2)}$ smaller than the 1/e^2^ diameter, we end up with a diffraction limited FWHM between 0.53 and 0.72 µm. Within the experimental uncertainties, the theoretical considerations and the experimental findings match. The glass thickness of the chromium line target was 1.5 mm which is much thicker than the 0.17 mm for which the objective is corrected for. In the experiments, microscope slides with a thickness of 1 mm, comparable to the thickness of the chromium line target, were used. We therefore expect the excitation spot diameter in the experiments to be similar to the spot diameter determined above.

### 3.2 Multimodal imaging of a blood smear

In Fig. 3

**Fig. 3:**
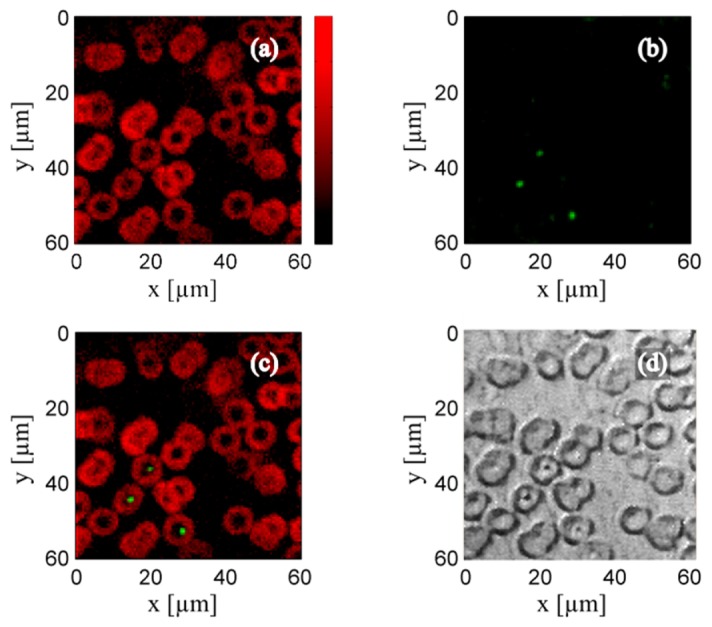
fOR-PAM image (a) and simultaneously obtained luminescence image (b) of human red blood cells. (c) Overlay of the photoacoustic and the luminescence image. (d) Bright-field image of the same region.

, we present photoacoustic and fluorescence measurements of an unstained blood smear. The blood smear was air-dried and then fixed with methanol on a silanized microscope slide. The excitation laser was modulated with a frequency of 10 MHz and the mean laser power before the objective lens was ~10 mW. Photoacoustic and fluorescence images were recorded simultaneously. The images consist of 101 × 101 pixels measured on an area of 60 µm × 60 µm. The image acquisition time was 300 s. In the fOR-PAM image shown in Fig. 3(a), the donut shape of the red blood cells (RBC) can be clearly seen. In Fig. 3(b), the luminescence image is shown. Three bright luminescence spots can be identified. The comparison of the overlay of the photoacoustic and luminescence images (Fig. 3(c)) with the bright-field microscopy image (Fig. 3(d)) reveals that these luminescent spots correspond to structures in the centers of the corresponding red blood cells. Red blood cells and other blood components usually do not exhibit fluorescence. Possibly, the fluorescence could stem from erythrocyte encapsulations, e.g. from Heinz bodies. It is known that Heinz bodies, which are aggregates of cytoplasmic degradation, exhibit fluorescence [29]. However, we cannot exclude that the fluorescence stems from contaminations or fixation artifacts. E.g., it is known that some fixatives can produce fluorescence in red blood cells [30]. Although we are not sure about the origin of the fluorescence, this result demonstrates the importance of multimodal imaging, as these structures are not visible in the photoacoustic image while fluorescence imaging is blind for the erythrocytes. In the bright-field microscopy image, one can observe both structures, but has no information on their chemical composition. Only the combined information obtained by photoacoustic and fluorescence imaging reveals their chemical difference.

## 4. Theoretical comparison of frequency domain OR-PAM vs. time domain OR-PAM

In the following, we will derive a formula to estimate the expected signal-to-noise ratio (SNR) of fOR-PAM with sinusoidal modulation in comparison with time domain PAM with pulsed excitation. In general, effective generation of photoacoustic signals requires the thermal and stress confinement conditions to be fulfilled [31]. For a resolution of 750 nm, as reported in section 3.1, the fulfillment of the thermal confinement requires a pulse length of about 1 µs or less and is therefore fulfilled for typically employed ns-laser pulses and also for modulated excitation in the MHz region. The stress confinement, on the other hand, requires pulse lengths shorter than 500 ps. The stress confinement condition is, therefore, neither fulfilled for the sinusoidal excitation nor for typically employed laser pulses with pulse lengths of several nanoseconds. Violation of the stress confinement condition leads to a blurring of the photoacoustic signals [31]. The bandwidth of the photoacoustic signals is then determined by the finite excitation pulse, rather than by the object. Therefore, in the following, we assume the excited volume to be small compared to the stress region and follow the formalism of Calasso et al. [32] for a point-like photoacoustic source. For an optical pulse with a time dependent intensity of the form $I(t)=\frac{E}{\text{θ}}f(t/\text{θ})$, with *E* being the fluence, *t* the time, and θ being a pulse width parameter. The resulting pressure is given as [32]:

$$p(t)=\frac{\text{σ}\;{\text{β}}_{1}}{4\text{π}\;c_{\text{p}}\;r}\cdot \frac{E}{{\text{θ}}^{2}}\cdot \frac{\text{d}}{\text{d}\hat{\tau }}f(\hat{\tau }).$$ (3)

Here β_1_ is the first coefficient in the power series expansion of the thermal expansion coefficient, σ is the optical cross section of the imaged particle, *c*_p_ is the specific heat capacity, *r* is the distance between source and detector, and $\hat{\tau }=\frac{t-r/c}{\text{θ}}$, with *c* being the speed of sound. In the following, we omit the coefficient σβ_1_/4π*c*_p_*r*, as we are only interested in the comparison between pulsed and modulated excitation, and not in quantitative values. For pulsed excitation, we assume a pulse of Gaussian shape:

$$I_{\text{G}}(t)=\frac{E_{\text{G}}}{\tau_{\text{G}}\sqrt{\text{π}}}\exp\left(-\frac{t^{2}}{{\tau_{\text{G}}}^{2}}\right),$$ (4)

where *E*_G_ is the corresponding fluence and $2\tau_{\text{G}}$is the pulse duration defined for a 1/e intensity decrease. We then find the time dependent pressure for a Gaussian pulse, *p*_G_(*t*), and its maximum, *p*_G,max_ = max{*p*_G_(*t*)}, to be:

$$p_{\text{G}}(t)\propto \frac{2tE_{\text{G}}}{{\tau_{\text{G}}}^{3}\sqrt{\text{π}}}\exp\left(-\frac{t^{2}}{{\tau_{\text{G}}}^{2}}\right).$$ (5a)

$$p_{\text{G,}\max}\propto \sqrt{\frac{2}{\text{e}\;\text{π}}}\frac{E_{\text{G}}}{{\tau_{\text{G}}}^{2}}.$$ (5b)

For the case of fOR-PAM we assume sinusoidal modulation with an intensity of the form $I_{\text{S}}\left(t\right)=I_{0}\left(1+\sin\left(2\text{π}\;f_{\text{S}}\;t\right)\right)$, where *I*_0_ is the mean optical intensity and *f*_S_ is the modulation frequency. We can rewrite the intensity as:

$$I_{\text{S}}\left(t\right)=\frac{E_{\text{S}}}{2\text{π}\;\tau_{\text{S}}}\left(1+\sin\left(t/\tau_{\text{S}}\right)\right),$$ (6)

With$\tau_{\text{S}}=1/(2\text{π}\;f_{\text{S}})$and $E_{\text{S}}=I_{0}/f_{\text{S}}$.

Using Eq. (3) we find the generated pressure *p*_S_(*t*) and its maximum *p*_S,max_ thereof as:

$$p_{\text{S}}(t)\propto 2\text{π}\;f_{\text{S}}^{2}E_{\text{S}}\cos\left(2\text{π}\;f_{\text{S}}t\right)=2\text{π}\;I_{0}f_{\text{S}}\cos\left(2\text{π}\;f_{\text{S}}t\right).$$ (7a)

$$p_{\text{S,max}}\propto 2\text{π}\;I_{0}\;f_{\text{S}}$$ (7b)

Equation (7) holds for the case of 100% modulation depth, i.e. if *I*_S_(*t*) fulfills Eq. (6). If the modulation laser has a cw offset, *I*_cw_, then the mean modulation intensity *I*_0_ can be calculated by subtracting the cw offset, *I*_cw_, from the total mean intensity: *I*_0_ = *I*_0,t_ – *I*_cw_. The total mean intensity, *I*_0,t_, can be experimentally determined via a power-meter.

Finally, we can calculate the ratio of the maximum generated pressure values for pulsed and sinusoidal excitation by dividing (5b) by (7b):

$$\frac{p_{\text{G},\max}}{p_{\text{S},\max}}=\sqrt{\frac{1}{2\text{π}\;\text{e}}}\cdot \frac{1}{\text{π}}\cdot \frac{E_{\text{G}}}{I_{0}}\cdot \frac{1}{{\tau_{\text{G}}}^{2}\cdot f_{\text{S}}}\approx \frac{1}{4\text{π}}\cdot \frac{E_{\text{G}}}{I_{0}}\cdot \frac{1}{{\tau_{\text{G}}}^{2}\cdot f_{\text{S}}}.$$ (8)

In practice, we are more interested in the obtained SNRs, rather than in the generated pressures. In the following we compare the expected SNR for a pulsed laser source with a 1/e intensity pulse length of 4 ns to sinusoidal excitation in the MHz region. For a pulse length of 4 ns, the generated acoustic frequencies spread deep into the GHz region. Detection of such high frequencies poses several problems, like acoustic dispersion and acoustic attenuation [33]. Usually measurement of the acoustic waves is done at considerably lower frequencies, typically below 100 MHz [34]. In the following we neglect acoustic attenuation, but include the frequency response of a typical ultrasonic transducer into the model. For this, we measured the response of an immersion transducer (Panametrics V358-SU, center frequency 50 MHz, 40 MHz FWHM) and calculated the convolution of the measured transducer response with the calculated pressure generated by the ns pulse. As result, we find that the measured response is broader than the pressure response given by Eq. (5a) and that the maximum measured signal is about a factor of 20 lower than when neglecting the transducer response. We thus introduce a factor ξ which describes the influence of the transducer on the maximum measured pressure. The SNR for the pulsed excitation is then SNR_G_ = *p*_G,max_⋅ξ / NEP, with NEP being the noise equivalent pressure. In our case ξ was estimated to be 1/20. On the other hand, by using a lock-in technique, the SNR can be considerably improved in case of sinusoidal modulation. As the noise increases with the square root of the bandwidth, we estimate the improvement in SNR by

$$\text{χ}=\sqrt{\frac{B_{\text{Det}}}{B_{\text{LIA}}}},$$ (9)

where *B*_Det_ is the ultrasonic detection bandwidth and *B*_LIA_ is the bandwidth of the lock-in detection, which depends on the integration time of the LIA, τ_LIA_ [35]:

$$B_{\text{LIA}}=\frac{1}{2\text{π}\;{\text{τ}}_{\text{LIA}}}.$$ (10)

The SNR for the sinusoidal excitation is then given by SNR_S_ = *p*_s,max_⋅χ / NEP. Finally, we assume the NEP to be the same for both methods and find the ratio of the SNRs of pulsed excitation measured in time domain to sinusoidal excitation measured via a lock-in technique to be:

$$\frac{{\text{SNR}}_{\text{G}}}{{\text{SNR}}_{\text{S}}}=\frac{p_{\text{G},\max}\cdot \text{ξ}}{\text{NEP}}\cdot \frac{\text{NEP}}{p_{\text{S},\max}\cdot \text{χ}}\approx \frac{1}{4\text{π}}\cdot \frac{E_{\text{G}}}{I_{0}}\cdot \frac{1}{{\tau_{\text{G}}}^{2}\cdot f_{\text{S}}}\cdot \frac{\xi }{\text{χ}}.$$ (11)

In Table 1

**Table 1:** SNR ratios for 4ns pulsed excitation and sinusoidal modulation

*f*_S_	τ_LIA_	SNR_G_/SNR_S_		SNR_S_/SNR_G_
5 MHz	10 ms	45		0.022
100 MHz	10 ms	2.3		0.44
100 MHz	1 ms	1.3		0.78

, we calculated SNR_G_/SNR_S_ (and its reciprocal value) for different experimental values of the modulation frequency and lock-in integration times. For this we assumed that in both excitation methods the limits of the ANSI safety standard are met. In case the maximum permissible exposure (MPE) limits are met, photo-damage should be low and the thermal stresses should be comparable for both measurement methods. The MPE values for skin and a wavelength of 405 nm are 200 J/m^2^ for pulsed excitation and $1.1\cdot 10^{4}\times {(\text{τ/sec)}}^{0.25}\;{\text{J/m}}^{\text{2}}$ for sinusoidal excitation, where τ is the exposure time in seconds [36]. The corresponding maximum intensity in case of sinusoidal modulation, *I*_0,max_, is then given by

$$I_{0,\max}=1.1\cdot 10^{4}\times {(\text{τ/sec)}}^{-0.75}\;{\text{W/m}}^{2}.$$ (12)

For the calculation we assumed ξ to be 1/20, as estimated before for the Panametrics V358-SU transducer, and the detection bandwidth to be two times the central frequency of the transducer, i.e. to be 100 MHz. We further assumed that the scanning speed is matched to the integration time of the LIA, i.e. that the dwell time, τ, on a single measurement point is equal to the lock-in integration time, τ_LIA_. For typical values of the modulation frequency of 5 MHz and an integration time of 10 ms, the SNR of the pulsed excitation is about 45 times better than for sinusoidal excitation if the MPE limits are met. The SNR of the sinusoidal case can be improved by using faster laser modulation. With an excitation frequency of 100 MHz, the SNR is expected to be only about a factor of two lower than for the pulsed excitation. A further improvement of the SNR ratio in favor of the sinusoidal modulation is possible by reducing the integration time and speeding up the scan, as SNR_S_ is directly proportional to the laser intensity but only inversely proportional to the square root of the bandwidth of the lock-in detection, *B*_LIA_. From Eqs. (9)–(12) we find that a reduction of the integration and dwell times, τ_LIA_ respectively τ, by a factor of *x* improves SNR_S_ by *x*^0.25^. Reducing the integration time to 1 ms while keeping the excitation frequency of 100 MHz leads to an SNR_S_ comparable to pulsed excitation. We note that our calculation does not include the transducer characteristics for the case of sinusoidal excitation. If modulation in the lower MHz region is used, one could employ transducers with a lower center frequency. Such transducers are available with larger element sizes, which have a better voltage to pressure response. By using a transducer with a larger element size, the estimation is shifted in favor of the sinusoidal modulation. Additionally, for the sinusoidal modulation one could use resonant ultrasonic transducers without backing. These transducers have a lower detection bandwidth, but therefore, a larger Q-factor and deliver higher output around the center frequency. Using a resonant transducer could improve the SNR in the case of sinusoidal modulation significantly.

## 5. Conclusion and outlook

In the present paper, we introduced simultaneous frequency domain optical-resolution photoacoustic and fluorescence microscopy using a continuous wave diode laser, which output power was sinusoidally modulated in the low MHz region. Photoacoustic and fluorescence signals were simultaneously measured with lock-in amplifiers. With the help of a photoacoustic image of a chromium line target, we determined the FWHM of the excitation spot to be approximately 750 nm. Simultaneous photoacoustic and fluorescence imaging was demonstrated on an unstained blood smear. In the photoacoustic image, the individual red blood cells could be identified on the basis of their donut-like form. In the luminescence image, three bright spots originating from the RBCs centers are visible. These three bright spots correspond to structures which are also visible in the bright-field microscopy image. However from the bright-field microscopy image, one cannot conclude that these three spots have a chemical composition different from the RBCs. By simultaneous photoacoustic and fluorescence imaging we were able to distinguish between these chemically different structures in the blood smear. Blood components of a healthy human do normally not exhibit fluorescence. In case of certain hemoglobinopathies erythrocyte inclusions, like Heinz bodies, basophilic stipplings, or Howell-Jolly bodies, can be found. For diagnosis and treatment, the correct classification of such red blood cell inclusion is essential. It is known that at least one of these inclusions, the so called Heinz bodies exhibit luminescence [29]. Heinz bodies occur e.g. in case of thalassemia but also as effect of chronic liver disease, after splenectomy [37], or due to mineral deficiency [38]. We therefore believe that the presented microscope is well suited for investigations in this field of hematology.

Additionally, we derived a formalism to compare the signal-to-noise ratios expected for pulsed photoacoustic excitation measured in time domain with sinusoidal PA excitation measured in frequency domain. We conclude that if one has to meet the maximum permissible exposure limits, pulsed excitation has a better SNR compared to sinusoidal excitation in the low MHz region. When increasing the modulation frequency to around 100 MHz, equivalent signal-to-noise ratios could be achieved. By using resonant transducers without backing, we think that the SNR of fOR-PAM could even exceed the SNR of classic OR-PAM using pulsed excitation.

During our experiments we observed a nearly constant SNR as a function of the modulation frequency, *f*_S_, between 4 MHz and 10 MHz. From Eq. (7b) we know that the photoacoustic pressure in the case of sinusoidal excitation is proportional to the modulation frequency. This suggests that higher modulation frequencies create higher photoacoustic pressures. However, in the frequency range between 4 MHz and 10 MHz, the decrease in laser modulation depth of the LuxX + diode laser cancels the increase of photoacoustic pressure. Therefore the hydrophone’s sensitivity, which is nearly constant between 4 MHz and 10 MHz, leads to a nearly constant SNR within this frequency region.

In order to increase the SNR as a function of modulation frequency, we plan to exchange the present excitation laser with a model capable of sinusoidal modulation up to 100 MHz. Such fast diode lasers are available below 15 kEUR. The laser sources for pulsed excitation normally consist of a pulsed Q-switched laser, e.g. a Nd:YAG laser, together with an optical parametric oscillator (OPO) or a dye laser [39–41]. Altogether, a typical laser system for pulsed excitation is more expensive than a fast diode laser plus ultrasound detection unit, i.e. lock-in amplifier and ultrasound transducer, for 100 MHz.

In the case of pulsed excitation, the OPO or the dye laser is necessary in order not to be limited to certain wavelengths as Q-switched ns lasers typically emit 1064 nm or 532 nm. Diode lasers emit at single wavelengths only, but they exist for many wavelengths from the near ultra-violet to the near infra-red. On average there exists a laser line every 20 nm between 375 nm and 830 nm, for the laser model used in this paper. The FWHM of the absorption spectrum of hemoglobin is around 30 nm. Such broad absorption bands are typical for molecules. Hence, nearly every molecule that absorbs in the visible spectral region can be potentially photoacoustically investigated by means of diode lasers.

Finally we address the issue that very high excitation intensities were used in our experiments. For the chromium target (Fig. 2) as well as for the blood smear (Fig. 3), intensities in the order of 10 GW/m^2^ were used. The MPE value for 10 ms integration time is several orders of magnitude lower, namely around 0.35 MW/m^2^. The MPE values given in this paper should ensure to avoid photodamage if human skin is exposed to laser light, but do not necessarily have to be fulfilled for microscopic samples like blood smears or microtome cuts. Microscopic samples are only a few µm thick and possibly have a absorption coefficient different from human skin. However, with the employed intensities we did frequently observe photo-bleaching during our experiments. For the following considerations, we will describe photo-bleaching by a single exponential decay rate, *k*, where *k* scales with *I^m^*, with *m*>2 [42,43]. The photoacoustic signal in frequency domain, PA_S_, as a function of exposure time, *t*, at a certain position, e.g. a single pixel, is proportional to the excitation intensity (compare with Eq. (7)) times the exponential decay caused by photo-bleaching:

$${\text{PA}}_{\text{S}}(t)\propto I_{0}\;{\text{e}}^{-k(I_{0})\;t}$$ (13)

From Eq. (13) we see that short integration times are desirable, as the photoacoustic signal bleaches exponentially with time. However, integration times are experimentally limited by the scanning speed or the lock-in detection. In order to maximize the photoacoustic signal, one has to optimize the intensity for a certain integration time. At low intensities, the photoacoustic signal, PA_S_, increases linearly with intensity. At a certain intensity *I*_max_, the photoacoustic signal becomes maximal. Increasing the intensity further, leads to a decrease of the photoacoustic signal, because photobleaching scales with *I*^m^. Therefore we expect that by increasing the modulation frequency to 100 MHz we can increase the SNR and reduce the effect of photo-bleaching simultaneously.
